# Mental-Health-Related Temporary Work Disability Among Informal Caregivers During the COVID-19 Lockdown in Spain (March–June 2020): A Nationwide Occupational Health Study

**DOI:** 10.3390/jcm15103746

**Published:** 2026-05-13

**Authors:** Eva María Gutiérrez Naharro, José Fernández Sáez, Raquel Ayuso Margañon, Ana María Montserrat Gala, José Ponce Blandón, Amalia Sillero Sillero

**Affiliations:** 1 Nursing Department, Escola Universitària Gimbernat, Adscrita a Universitat Autònoma de Barcelona, 08174 Sant Cugat del Vallès, Spain; egutinaharro@gmail.com (E.M.G.N.); amalia.sillero@eug.es (A.S.S.); 2 Facultat d’Infermeria, Campus Terres de l’Ebre, Universitat Rovira i Virgili, 43500 Tarragona, Spain; 3 Servei d’Atenció Primària Terres de l’Ebre, Institut Català de la Salut, 43500 Tarragona, Spain; 4 Unitat de Suport a la Recerca Terres de l’Ebre, Fundació Institut Universitari per a la Recerca a l’Atenció Primària de Salut Jordi Gol i Gurina (IDIAPJGol), 43500 Tarragona, Spain; 5 Social Determinants and Health Education Research Group (SDHEd), Hospital del Mar Research Institute, 08003 Barcelona, Spain; 6 Hospital del Mar Nursing School (ESIHMar), Universitat Pompeu Fabra-Affiliated, 08003 Barcelona, Spain; 7 Department of Medicine, Autonomous University of Barcelona (UAB), 08193 Barcelona, Spain; anamaria.montserrat@uab.cat; 8 Nursing Department, Faculty of Nursing, Physiotherapy and Podiatry, University of Seville, 41009 Seville, Spain; japonce@us.es

**Keywords:** informal caregivers, temporary work disability, mental health, occupational health, COVID-19 lockdown

## Abstract

**Background/Objectives**: During the first COVID-19 lockdown, the sudden disruption of formal care services substantially increased reliance on informal caregiving. Emerging evidence suggests that increased caregiving demands may have contributed to a higher burden of mental-health-related temporary work disability; however, population-based data from occupational health systems remain limited. This study aimed to quantify and characterise, descriptively, the sociodemographic, clinical, and territorial characteristics of mental-health-related temporary work disability among workers with informal caregiving responsibilities in Spain during the first COVID-19 lockdown, and to descriptively examine differences between episodes occurring among workers with and without caregiving responsibilities. **Methods**: A retrospective descriptive study was conducted using anonymised nationwide occupational health records from Mutua Asepeyo. All episodes of temporary work disability certified for mental and behavioural disorders (ICD-10 F00–F99) between 14 March and 21 June 2020 were analysed. Caregiver status was determined based on documented informal caregiving responsibilities recorded within the occupational disability records. Sociodemographic, occupational, clinical, and territorial variables were examined using descriptive statistics and non-parametric tests. **Results**: A total of 2857 caregiver-associated episodes were identified, representing 55.6% (95% CI: 54.2–57.0) of all mental-health-related temporary work disability episodes during the study period. The majority involved women (68.1%) and caregivers of older dependent adults (59.3%). Generalised anxiety disorder was the most frequent diagnosis, followed by adjustment disorders and acute stress reactions, with significant differences by sex and employment regime. Marked territorial variability was observed, as well as longer durations of temporary work disability in specific regions and among women. **Conclusions**: A substantial proportion of mental-health-related temporary work disability episodes during the lockdown occurred among workers with informal caregiving responsibilities, particularly women and those caring for older dependents. These findings suggest that informal caregiving may be a determinant of occupational mental health during crises. However, given the descriptive and unadjusted nature of the study, no causal inferences can be drawn. Further research is needed to understand these associations better and inform future occupational health strategies.

## 1. Introduction

Informal caregiving is a cornerstone of global health systems, with 17–20% of European adults providing unpaid care to dependents [1,2,3]. These unpaid contributions account for up to 80% of total long-term care hours, forming a substantial component of long-term care provision across healthcare systems. Despite their social and economic relevance, informal caregivers often experience exhaustion, emotional strain, and elevated risks of anxiety and depression, particularly where formal support systems are limited [4,5,6].

The COVID-19 pandemic exacerbated these vulnerabilities. The World Health Organisation (WHO) declared a global health emergency in March 2020 [7], triggering lockdowns, mobility restrictions, and the closure of community and health services [8]. Although essential to limit transmission, these measures disrupted social networks and formal care supports, imposing additional care responsibilities on families [9]. During this period, the suspension of these services disproportionately increased women’s caregiving responsibilities, as they are more likely to assume informal caregiving roles within households.

Longitudinal studies across Europe demonstrated substantial increases in anxiety and depressive symptoms, particularly among caregivers [10,11]. Many individuals assumed caregiving roles suddenly and without preparation, resulting in increased emotional burden [12]. Caregivers of people with dementia or disabilities experienced severe isolation, moral distress, and exhaustion following the interruption of day centres and respite services [13,14]. The mental health impact was consistently observed across countries, particularly in the form of anxiety, depressive symptoms, and sleep disturbances among informal caregivers [15]. In Spain, the study period analysed here corresponds to the officially defined first COVID-19 lockdown (14 March to 21 June 2020), including both the strict confinement phase and the initial de-escalation period.

From a clinical perspective, mental health in occupational settings is commonly operationalised through internationally standardised diagnostic categories. In Spain, mental-health-related temporary work disability (TWD) is certified according to the ICD-10 classification of mental and behavioural disorders, which includes anxiety and depressive disorders, adjustment-related conditions and acute stress reactions. These diagnostic groups have been widely documented as predominant forms of psychological distress during the COVID-19 pandemic, particularly among caregivers and women [10,15]. Incorporating this clinical framework helps contextualise how pandemic-related caregiving demands intersect with recognised mental health conditions in the labour environment.

Spain, with its familialist care model, provides a paradigmatic example. The suspension of educational and community services and high infection rates disproportionately affected women who traditionally assume a greater share of informal caregiving responsibilities, particularly in elder care [16,17]. National institutional reports documented the emotional and socioeconomic impact of the pandemic on informal caregivers, underscoring the strain placed on family-based care arrangements during the crisis [18]. In parallel, workers in the services sector and in health and social care were particularly exposed to increased psychosocial demands during the pandemic, as occupational pressure frequently coexisted with intensified caregiving responsibilities at home. During the lockdown period, limitations in the provision of formal health and nursing care were also widely reported, including reduced accessibility, continuity, and availability of routine support services [19]. Beyond their impact on healthcare professionals themselves, these disruptions altered usual patterns of care delivery in the community, reducing professional support that often complements informal caregiving under non-crisis conditions. For individuals providing unpaid care at home, loss or reduction in this routine professional support may have exacerbated caregiving demands and contributed to increased psychological strain [20].

Previous studies have shown that disruptions in formal care provision during the COVID-19 pandemic were closely intertwined with changes in informal caregiving arrangements, with reductions in formal care associated with increased strain and adverse psychological outcomes among care recipients and caregivers. However, these relationships have rarely been examined from an occupational health perspective or using labour-related outcomes [21].

Despite this growing evidence of caregiver distress, limited research has examined the functional consequences in the labour sphere, particularly in relation to mental-health-related temporary work disability (TWD). While recent studies conducted in Spain and other European countries have identified gender and psychosocial factors associated with sickness absence during the pandemic [22], most analyses have focused on the general working population rather than on situations specifically compatible with informal caregiving responsibilities. In this context, these dynamics are likely shaped not only by caregiving burden itself, but also by gendered role expectations, employment conditions, and regional differences in the availability of formal and informal support. From a conceptual perspective, the intersection between informal caregiving and occupational health can be understood within frameworks of role strain and work–care conflict. These frameworks describe how competing demands between unpaid caregiving and employment responsibilities may generate psychosocial stress, emotional overload, and functional impairment. This conceptual lens is particularly relevant in crisis contexts such as the COVID-19 lockdown, where reductions in formal support intensified the simultaneous demands placed on workers with caregiving responsibilities.

In parallel, occupational health professionals reported atypical patterns of sickness absence among workers with substantial caregiving duties during the lockdown period. Some episodes appeared to occur in contexts of intensified non-work demands, including caregiving responsibilities, coinciding with increased psychological distress and functional impairment. International literature has documented similar patterns of mental-health-related sickness absence in contexts of excessive non-work demands, particularly caregiving during the pandemic [23,24]. Related phenomena have been described in the literature using various terms to capture the interaction between caregiving burden and sickness absence. However, these patterns remain insufficiently examined using standardised occupational health data.

In this study, we refer descriptively to episodes of mental-health-related TWD occurring in workers with documented informal caregiving responsibilities, without implying causality or proposing a new category. These episodes are interpreted as coinciding with increased caregiving demands during the lockdown, rather than as evidence of any specific mechanism or misuse of sickness absence.

To address this evidence gap, the present study aimed to descriptively quantify and characterise the sociodemographic, clinical, occupational, and territorial characteristics of mental-health-related TWD among workers with informal caregiving responsibilities during the first COVID-19 lockdown in Spain, and to compare these episodes with those occurring in non-caregivers.

## 2. Materials and Methods

### 2.1. Study Design and Setting

This was a retrospective descriptive study based on routinely collected occupational health records from Mutua Asepeyo, one of the largest mutual insurance entities collaborating with the Spanish Social Security system and authorised to manage occupational contingencies, including temporary work disability, under public regulation. The analysis forms part of a broader research programme examining mental-health-related temporary work disability (TWD) during the COVID-19 pandemic in Spain [17]. However, the present work constitutes an independent secondary analysis explicitly focused on informal caregiving and patterns occurring in the context of documented informal caregiving responsibilities.

The study period covered the national lockdown and the immediate post-lockdown phase, from 14 March to 21 June 2020. During this period, access to community services, educational institutions, and formal care resources was markedly reduced, substantially increasing reliance on family-based care.

This design enabled a descriptive characterisation of mental-health-related TWD episodes among workers with documented informal caregiving responsibilities. The study does not involve longitudinal follow-up and does not aim to establish causal relationships; all comparisons are exploratory and unadjusted.

### 2.2. Participants

The study population included insured workers aged 18 years or older who experienced at least one episode of temporary work disability certified for mental and behavioural disorders (ICD-10 codes F00–F99) during the study period.

Episodes were included when occupational health records documented informal caregiving responsibilities, defined as unpaid care provided to dependent relatives (children, partners, parents, or other dependent adults). This information derives from a routinely collected administrative field completed during the occupational health clinical interview, in which workers indicate whether they provide unpaid care for a dependent family member. This field is part of the standard clinical and administrative documentation gathered during sickness-absence assessments and is recorded consistently across Asepeyo centres. As this is an administrative rather than a psychometric measure, it captures the presence of caregiving responsibilities for contextual and occupational purposes and does not constitute a validated instrument. Caregiving responsibilities may therefore be underreported if not explicitly recorded during the occupational health assessment.

In this study, informal caregivers refer exclusively to unpaid, non-professional care provided to dependent family members. This definition explicitly excludes professional or paid caregiving roles, such as home-care workers, personal assistants, or employees in health and social care services. This study, therefore, focuses exclusively on informal caregiving roles, as defined in administrative occupational health records.

Temporary work disability (TWD) and informal caregiving are two independent constructs in the administrative dataset. TWD is a certified labour-administrative outcome based solely on ICD-10 mental health diagnoses, whereas informal caregiving refers to unpaid care responsibilities recorded in a separate administrative field. These variables are not linked in the registry and were analysed descriptively for their concurrent occurrence during the lockdown.

Episodes were excluded if: (1) the sickness absence was initiated before 14 March 2020; (2) the primary diagnosis corresponded to non-psychiatric causes; or (3) key sociodemographic or clinical information was missing.

### 2.3. Variables

The primary outcome variable was the duration of temporary work disability (TWD), measured in days as a continuous variable.

Episodes occurring in the context of documented informal caregiving responsibilities were identified descriptively. This descriptive classification does not represent a diagnostic or administrative category but was used analytically to characterise patterns of mental-health-related TWD coinciding with informal caregiving responsibilities. These were defined as mental-health-related TWD episodes occurring during the lockdown period in workers with documented informal caregiving responsibilities for dependent relatives. No inference regarding the appropriateness of sickness absence was made.

The following independent variables were analysed: sex, age, autonomous community of residence, employment regime (general vs. self-employed), primary mental health diagnosis (ICD-10), presence of chronic disease, and prior psychiatric history.

Caregiving responsibilities were classified by the type of dependent person (older adult, child/adolescent, or other dependent adult), as no information was available on dependency level, clinical condition, or caregiving intensity.

### 2.4. Statistical Analysis

All analyses were performed using IBM SPSS Statistics version 27.0 (IBM Corp., Armonk, NY, USA). Continuous variables were summarised using means and standard deviations (SD), as measures of central tendency and dispersion. Categorical variables were described using absolute frequencies and percentages.

Comparisons between groups (episodes with documented caregiving responsibilities vs. those without) were performed using: the χ^2^ test (for categorical variables with two categories); the Z test for differences in proportions for categorical variables with more than two categories, including autonomous communities; and the non-parametric Mann–Whitney U test for continuous variables. A 95% confidence interval was calculated for key proportions to quantify precision.

All analyses were conducted for descriptive and exploratory purposes. Statistical tests were two-tailed, and *p*-values < 0.05 were considered statistically significant.

### 2.5. Ethical Considerations

This study was conducted in accordance with the Declaration of Helsinki (2013 revision) [25] and was approved by the Asepeyo Institutional Research Ethics Committee (Ref. 2022/48-MLA-ASEPEYO, June 2022). All data were fully anonymised, and individual consent was waived under Spanish and EU data-protection regulations (Regulation [EU] 2016/679 and Organic Law 3/2018) [26], as the study relied exclusively on secondary, non-identifiable data.

## 3. Results

**Figure 1:**
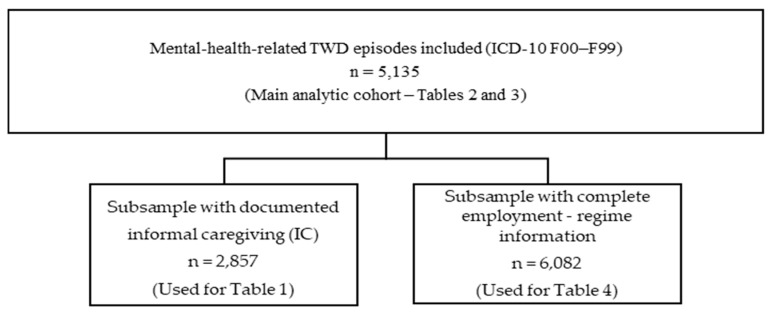
Flow diagram of analytic subsamples.

Different analytic subsamples were used depending on the research question and the availability of specific variables. Because not all variables were available for all episodes in the administrative dataset, analyses were conducted using the largest possible subsample with complete information for each specific comparison. Table 1 presents only episodes involving documented informal caregiving responsibilities (n = 2857). Table 2 and Table 3 include all mental-health-related TWD episodes identified during the study period (n = 5135), as these comparisons required information available for the full cohort. Table 4 includes all TWD episodes with complete employment-regime data (n = 6082), as this variable is routinely recorded for all insured workers regardless of caregiving status or the clinical cause of the disability, which explains why this subsample exceeds the cohort of mental-health-related TWD episodes. A flow diagram has been added to clarify the derivation of each analytic subsample (Figure 1).

### 3.1. Sociodemographic and Occupational Characteristics

A total of 2857 mental-health-related TWD episodes occurring in workers with documented informal caregiving responsibilities met the inclusion criteria. The mean age was 47.2 years (SD 9.5), and 68.1% were women. Most episodes involved workers in the services sector (34.2%) or in health and social care (29.6%).

Regarding caregiving responsibilities, 59.3% involved care for older adults, 27.8% for children or adolescents, and 12.9% for other dependent adults. Chronic disease was documented in 30.9% of cases, and 18.3% of workers had a prior psychiatric history (Table 1).

### 3.2. Prevalence and Territorial Distribution

Among all mental-health-related TWD episodes during the study period (n = 5135), 55.6% (95% CI: 54.2–57.0) occurred in workers with documented informal caregiving responsibilities. This proportion describes the relative weight of caregiving responsibilities among mental-health-related TWD episodes observed during the lockdown period and reflects the concentration of caregiving within this subgroup.

Because the occupational health database does not include the full denominator of insured workers with and without caregiving responsibilities, this proportion cannot be interpreted as a prevalence estimate of informal caregiving or as formal evidence of over-representation within the insured working population. European population-based studies indicate that approximately 17–20% of adults provide informal care [1,2,3]; however, these estimates are provided solely for contextual reference and do not constitute denominators derived from the administrative dataset. Within these constraints, this proportion remains informative for describing how mental-health-related TWD episodes during the lockdown were distributed across workers with and without documented caregiving responsibilities, and for examining territorial variability in this pattern.

Significant territorial variability was observed (Table 2). Higher proportions were identified in Cantabria (71.4%), La Rioja (66.7%), the Canary Islands (63.1%), and Catalonia (56.3%). Several autonomous communities showed statistically significant differences, indicating heterogeneous regional patterns.

For each autonomous community, the *p*-value corresponds to the comparison between workers with and without documented informal caregiving responsibilities, based on the proportions shown in the table.

**Figure 2:**
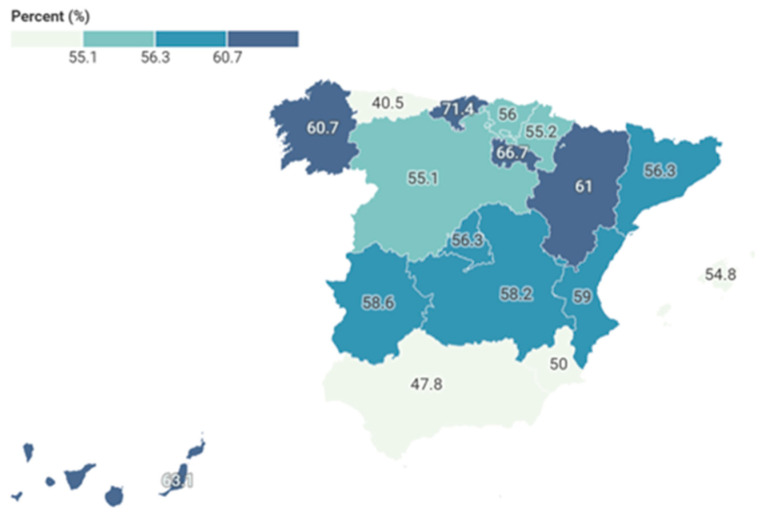
Quartile-based choropleth map of caregiving-related mental-health TWD episodes across autonomous communities. Note: Quantile classification is descriptive and does not imply statistical significance.

A choropleth map based on quartile classification (four categories) was created (Figure 2) to enhance the visual interpretation of territorial variability. This approach highlights the distribution of values across regions, with communities grouped into descriptive quartiles ranging from lower proportions of caregiving-related TWD (<45%) to higher proportions (≥60%), complementing the information in Table 2.

### 3.3. Mental Health Diagnoses by Sex (n = 5135)

Across all episodes, generalised anxiety disorder was the most frequent diagnosis (69.2%), with no significant difference between women and men (Table 3). However, adjustment disorders and acute stress reactions were significantly more frequent among women, whereas men showed slightly higher proportions of specific depressive diagnoses. Less frequent diagnoses (<2%) are not shown in the table for clarity.

### 3.4. Mental Health Diagnoses by Employment Regime

Differences emerged when diagnoses were stratified by employment regime (Table 4). While anxiety disorders predominated in both groups, self-employed workers showed significantly higher proportions of mild major depressive disorder and recurrent depressive disorder. No causal inference was drawn from these differences.

### 3.5. Mean Duration of Temporary Work Disability (Days) by Autonomous Community and Sex

The mean duration of TWD varied substantially by autonomous community, ranging from 36.3 days in Navarra to 59.1 days in Cantabria. Sex differences were observed in several regions, with women showing significantly longer durations in Galicia, Castile and León, and the Valencian Community (Table 5).

Table 5 includes only autonomous communities with enough TWD episodes to allow reliable estimation of mean duration values (≥20 episodes); communities with very small n were not presented due to unstable averages.

## 4. Discussion

This study describes the intersection between informal caregiving responsibilities and mental-health-related temporary work disability (TWD) during the first COVID-19 lockdown in Spain. Using nationwide occupational health records, the findings highlight how caregiving responsibilities, sex, employment regime, and territorial context are associated with distinct patterns of mental-health-related TWD.

As a retrospective descriptive analysis based on administrative data, the findings should be interpreted as observed patterns within the dataset and do not imply independent associations or causal relationships. They reflect the concurrence of caregiving demands and certified mental-health-related work disability during a period of major disruption of formal care services, within a structural context in which informal caregiving responsibilities are unequally distributed by gender.

### 4.1. Informal Caregiving and Mental-Health-Related Work Disability

More than half of all mental-health-related TWD episodes during the study period occurred among workers with documented informal caregiving responsibilities. This finding aligns with emerging evidence that informal care constitutes a relevant social and occupational determinant of mental health, particularly under crisis conditions [27,28,29]. The predominance of anxiety-related diagnoses is consistent with international research describing heightened uncertainty, emotional overload, and role conflict among caregivers during pandemic-related restrictions [30,31]. As highlighted by these studies, caregivers consistently show higher psychological vulnerability than non-caregivers during periods of increased care demands, which may help contextualise the high proportion observed in our cohort. Taken together, these findings suggest that mental-health-related TWD among caregivers occurred in contexts characterised by sustained caregiving demands and elevated psychological distress.

At the same time, the intersection between caregiving responsibilities and mental-health-related TWD is embedded in a wide range of everyday circumstances that shape how strain is perceived and managed. Differences in the intensity and organisation of care, in the availability of support within families, or in workers’ ability to reconcile caregiving with employment may help explain some of the observed variability. Considering these contextual elements adds interpretive depth to the descriptive patterns identified in this study. In this sense, mental-health-related work disability among caregivers may reflect not only the presence of caregiving responsibilities per se, but also contextual factors such as sustained vigilance, emotional responsibility, and uncertainty regarding the adequacy of available support during the lockdown period.

### 4.2. Sex Differences and Differential Exposure to Caregiving Demands

Women accounted for more than two-thirds of the TWD episodes occurring among workers with informal caregiving responsibilities. This finding should not be interpreted as an inherent gender difference, but rather as a reflection of structural gender inequalities in the distribution of unpaid care work, as consistently described in European contexts [32,33,34]. Such structural imbalances may increase exposure to psychosocial strain, particularly when caregiving demands coexist with occupational pressures, as observed during the COVID-19 lockdown.

From a broader perspective, these patterns can be interpreted within social determinants of health frameworks and models of work-care conflict, in which gendered divisions of unpaid care work shape differential exposure to stressors and associated mental health outcomes.

Although the overall prevalence of anxiety disorders was similar by sex, women exhibited significantly higher rates of adjustment disorders and acute stress reactions, suggesting differences in how caregiving-related stress is experienced and manifested.

These variations may also reflect how caregiving strain is perceived, expressed, and negotiated within families and workplaces, where gendered expectations often influence both emotional and practical caregiving roles. These patterns are consistent with literature reporting sex-specific responses to psychosocial stressors during periods of increased care demand [35].

In addition to differences in prevalence, women are more likely to be exposed to caregiving contexts characterised by higher emotional demands, greater continuity of care, and fewer opportunities for delegation or external support.

Taken together, these factors may contribute to the observed patterns of mental-health-related work disability and underscore the importance of incorporating sex- and gender-sensitive perspectives in occupational mental health research.

### 4.3. Eldercare and Intensified Care Demands

Care for older dependent adults was the most frequent caregiving context identified, accounting for nearly 60% of episodes. This finding is particularly relevant in the context of the widespread closure or reduction in day-care centres, residential services, and community support resources during the lockdown. Previous studies have reported higher psychological distress and reduced functional capacity among caregivers of older adults, particularly when formal supports are withdrawn [36,37]. In this context, the high prevalence of eldercare observed may be associated with the temporary disruption of formal support services, which shifted care responsibilities from institutional settings to households. From a conceptual perspective, caregiving in later life is highly heterogeneous, with substantial variation in dependency levels, intensity of care, and availability of formal and informal support. Such variability may contribute to differences in psychological burden and functional impact across caregiving contexts.

Qualitative research conducted during the pandemic has described experiences of isolation, sustained vigilance, and emotional strain among this group [38], which provides contextual support for the patterns of prolonged TWD observed in certain regions.

### 4.4. Employment Regime and Mental Health Vulnerability

Differences observed by employment regime suggest that self-employed caregivers experienced a distinct pattern of vulnerability, with higher proportions of depressive diagnoses. These findings should be interpreted cautiously, as the study design does not allow causal inference. Nonetheless, the results are consistent with earlier evidence suggesting that employment insecurity is associated with adverse mental health outcomes among caregivers, particularly during periods of social and economic instability [39]. In such contexts, the practical challenges of reconciling caregiving responsibilities with income-dependent or non-standard work arrangements may contribute to heightened levels of psychological strain among self-employed workers. Beyond the employment regime alone, broader structural factors such as financial insecurity, variable autonomy at work or unstable working conditions may interact with caregiving demands, potentially amplifying mental-health vulnerability among certain groups.

### 4.5. Territorial Variability

Marked territorial differences were observed in the prevalence and duration of TWD episodes among workers with informal caregiving responsibilities across autonomous communities. These differences likely reflect heterogeneity in demographic composition, regional care infrastructure, and the extent of service disruption during lockdown. Within a decentralised health and social care system such as Spain’s, territorial patterns may be influenced by regional differences in the availability of community care services, the organisation of social support systems, and variability in how formal care resources were disrupted. In addition, regional differences in how caregiving strain is recognised, communicated, or managed may contribute to the observed variability.

Similar regional variability has been reported in previous studies examining decentralised health and social care systems during emergencies [40,41]. From an epidemiological perspective, these findings reinforce the importance of considering regional context when analysing caregiving-related mental health outcomes. These territorial patterns should also be interpreted in light of broader contextual dynamics that cannot be fully captured in administrative datasets. Differences in help-seeking behaviours, workplace expectations or the perceived accessibility of services across regions may influence how mental-health-related TWD is recognised or formalised, adding interpretive nuance to the observed variability.

### 4.6. Chronic Disease, Psychiatric History, and Accumulated Vulnerability

A substantial proportion of caregivers experiencing mental-health-related TWD had documented chronic disease or prior psychiatric history, supporting the notion of accumulated vulnerability. Although information on psychiatric conditions among care recipients was not available, caregiving in the context of chronic mental health conditions is likely to be associated with sustained emotional demands, and prior psychiatric history among caregivers may reflect increased vulnerability in such caregiving situations. In practice, how individuals respond to overlapping stressors can differ considerably, and this variability may influence how chronic conditions interact with caregiving strain. Previous research indicates that caregiving stress may interact with pre-existing health conditions, contributing to prolonged symptom duration and delayed recovery [42]. Early identification of caregivers with documented vulnerabilities may be relevant for occupational health surveillance and prevention strategies, although further longitudinal research is required. These patterns should also be viewed within the broader context of cumulative life stressors, as the coexistence of chronic illness, caregiving responsibilities and work-related strain may enhance vulnerability in ways that descriptive data cannot fully disentangle.

### 4.7. Clinical and Occupational Health Implications

The findings have potential relevance for clinical and occupational health practice. Although this study does not assess healthcare system workload or economic costs directly, the observed concentration of mental-health-related temporary work disability among caregivers during the lockdown may help contextualise broader pressures on occupational health and social care systems during crises.

The high concurrence between caregiving responsibilities and mental-health-related TWD highlights informal caregivers as a group warranting increased attention in occupational mental health assessment. Mental-health-related TWD occurring in caregivers may reflect contexts of increased psychosocial strain during periods of reduced formal support.

Within healthcare systems, including nursing and occupational health services, previous research has highlighted the relevance of nurse-led approaches and coordinated occupational health strategies in the early detection and support of workers experiencing psychosocial strain [43,44]. Given the descriptive patterns identified in this study, these approaches should be viewed as potential areas for future investigation rather than as direct practice implications. Any translation of these findings into practice requires careful consideration of the diversity of caregiving situations within the workforce, as well as the multiple social and occupational factors that shape how psychological distress is recognised and managed [45,46]. Future research should explore targeted interventions aimed at mitigating caregiver-related mental health risks within occupational settings.

These findings reinforce the need to integrate social and caregiving contexts into occupational mental health surveillance frameworks, particularly in periods of systemic disruption.

### 4.8. Strengths and Limitations

The main strengths of this study include its large, real-world sample, nationwide scope, and use of routinely collected occupational health data, allowing for a comprehensive overview of caregiving-related mental health disability during an unprecedented period. This approach enables the identification of real-world patterns that might not emerge through survey-based approaches.

However, several limitations should be considered. Caregiving responsibilities were identified from self-reported occupational health records and did not capture their intensity, duration, or the number of dependents. As caregiving is recorded when reported or considered contextually relevant during the occupational health assessment, some degree of misclassification cannot be excluded. While under-ascertainment of informal caregiving responsibilities is possible, the direction of this potential bias cannot be determined with certainty. Because caregiving information is documented during mental-health-related temporary work disability assessments, workers presenting with psychological distress may be more likely to report or have caregiving responsibilities recorded than other workers. As a result, the observed proportion of mental-health-related TWD episodes occurring among caregivers could be either underestimated or overestimated. Information on informal support networks and caregiving hours was unavailable. No data were available on the characteristics of care recipients, which limits a more precise characterisation of caregiving burden and precludes the examination of caregiver–care recipient associations.

Additionally, the observational and cross-sectional nature of the analysis precludes causal inference. Finally, the findings may not be generalisable to uninsured, informal, or unemployed caregivers. As with all administrative datasets, the information available reflects the purpose for which it was collected, which may introduce heterogeneity that cannot be fully assessed.

Despite these limitations, the use of a large nationwide administrative dataset allows for a descriptive overview of patterns of mental-health-related temporary work disability occurring in the context of informal caregiving during the lockdown period. This approach offers insight into real-world occupational health dynamics that may be difficult to capture through smaller or self-selected samples.

Overall, the findings highlight the importance of considering caregiving-related contexts when examining occupational mental health, particularly during crisis situations in which non-work demands may substantially influence workers’ psychological wellbeing.

## 5. Conclusions

The first COVID-19 lockdown exposed the critical role of informal caregivers in sustaining daily life amid severe service disruptions. In this nationwide occupational health study, a substantial proportion of mental-health-related temporary work disability (TWD) episodes occurred among workers with documented caregiving responsibilities, particularly women, caregivers of older adults, individuals with pre-existing health conditions, and workers in more vulnerable employment contexts.

These findings support considering informal caregiving responsibilities as a relevant contextual factor in occupational mental health assessment and surveillance. Improved coordination among healthcare, occupational health services, and social care resources may help identify caregivers at risk of prolonged work disability, particularly during periods of system disruption.

Further research is warranted to evaluate targeted preventive and supportive interventions aimed at reducing mental-health-related work disability among informal caregivers in occupational settings.

## Figures and Tables

**Table 1:** Sociodemographic, occupational, caregiving, and clinical characteristics of workers with documented informal caregiving responsibilities (n = 2857).

Variable	n (%)/Mean (SD)	95% CI
Sociodemographic characteristics		
Age (years), mean (SD)	47.2 (9.5)	-----
Female n (%)	1947 (68.1)	66.4–69.9
Male n (%)	910 (31.9)	30.1–33.6
Occupational characteristics		
Health and social care, n (%)	846 (29.6)	27.9–31.3
Services, n (%)	978 (34.2)	32.5–36.0
Industry, n (%)	631 (22.1)	20.6–23.6
Education/Public administration, n (%)	402 (14.1)	12.8–15.3
Caregiving characteristics		
Older adult dependent, n (%)	1694 (59.3)	57.5–61.1
Child/adolescent dependent, n (%)	795 (27.8)	26.2–29.5
Other dependent adult, n (%)	368 (12.9)	11.7–14.1
Clinical characteristics		
Chronic disease, n (%)	884 (30.9)	29.2–32.6
Prior psychiatric history, n (%)	523 (18.3)	16.9–19.7

Note: Values are presented as n (%) unless otherwise indicated. 95% confidence intervals (95% CIs) are provided for proportions to quantify precision.

**Table 2:** Distribution of Mental-Health-Related Temporary Work Disability Episodes by Informal Caregiving Status and Autonomous Community (n = 5135).

Autonomous Community	Total n	Without Caregiving Responsibilities n (%)	With Caregiving Responsibilities n (%)	*p*-Value
Andalusia	561	293 (52.2)	268 (47.8)	0.136
Aragon	105	41 (39.0)	64 (61.0)	0.002
Asturias	116	69 (59.5)	47 (40.5)	0.004
Canary Islands	141	52 (36.9)	89 (63.1)	<0.001
Cantabria	35	10 (28.6)	25 (71.4)	<0.001
Castile and León	267	120 (44.9)	147 (55.1)	0.019
Castile-Mancha	79	33 (41.8)	46 (58.2)	0.039
Catalonia	2608	1140 (43.7)	1468 (56.3)	<0.001
Valencian	300	123 (41.0)	177 (59.0)	<0.001
Basque Country	250	110 (44.0)	140 (56.0)	0.007
Extremadura	29	12 (41.4)	17 (58.6)	0.189
Galicia	183	72 (39.3)	111 (60.7)	<0.001
Balearic Islands	42	19 (45.2)	23 (54.8)	0.383
La Rioja	42	14 (33.3)	28 (66.7)	0.002
Madrid	213	93 (43.7)	120 (56.3)	0.009
Murcia	68	34 (50.0)	34 (50.0)	1.000
Navarre	96	43 (44.8)	53 (55.2)	0.149

Note: Classification is based on documented informal caregiving responsibilities. Values are presented as frequencies and percentages. *p*-values correspond to within-community comparisons between workers with and without documented caregiving responsibilities.

**Table 3:** Mental Health Diagnosis by Sex (n = 5135).

Diagnosis	Total, n (%)	Women, n (%)	Men, n (%)	*p*-Value
Generalised anxiety disorder	3552 (69.17)	2252 (69.10)	1300 (69.30)	0.884
Adjustment disorder	589 (11.47)	351 (10.77)	238 (12.69)	0.038
Major depressive disorder, mild	386 (7.52)	236 (7.24)	150 (8.00)	0.324
Acute stress reaction	133 (2.59)	102 (3.13)	31 (1.65)	0.001
Neurotic depression	111 (2.16)	83 (2.55)	28 (1.49)	0.012

**Table 4:** Mental Health Diagnosis by Employment Regime (n = 6082).

Diagnosis	Total, n (%)	General Regime, n (%)	Self-Employed n (%)	*p*-Value
Generalised anxiety disorder	4282 (70.40)	3766 (71.03)	516 (66.15)	0.005
Adjustment disorder	689 (11.33)	606 (11.43)	83 (10.64)	0.516
Major depressive disorder, mild	422 (6.94)	331 (6.24)	91 (11.67)	<0.001

**Table 5:** Mean Duration of Temporary Work Disability (days) by Autonomous Community and Sex in Regions with ≥20 Episodes.

Autonomous Community	Total Mean (SD) *	Women Mean (SD) *	Men Mean (SD) *	*p*-Value
Cantabria	59.05 (29.65)	57.5 (28.8)	61.7 (31.9)	0.940
Aragon	51.22 (29.769)	50.4 (29.5)	55.1 (31.3)	0.456
Galicia	48.92 (28.65)	52.2 (27.8)	43.2 (29.4)	0.025
Madrid	47.07 (26.71)	47.8 (27.4)	45.8 (25.5)	0.671
Catalonia	40.95 (29.01)	41.6 (29.1)	40.1 (28.9)	0.113

* SD: standard deviation. Note: Values are presented as mean (standard deviation). Sex comparisons were performed using the Mann–Whitney U test.

## Data Availability

The data supporting the findings of this study are derived from anonymized nationwide occupational health records provided by Mutua Asepeyo. Due to legal and ethical restrictions related to data protection and the use of administrative health records, the underlying individual-level dataset cannot be made publicly available. Aggregated data and analytical summaries that support the results reported in this article are available from the corresponding author upon reasonable request and subject to approval by the data holder.

## Abbreviations

The following abbreviations are used in this manuscript:

WHO	World Health Organisation
OECD	Organisation for Economic Co-operation and Development
EU	European Union
TWD	Temporary Work Disability
ICD-10	International Classification of Diseases, 10th Revision
COVID-19	Coronavirus Disease 2019
SDGs	Sustainable Development Goals
